# Treatment of Anxiety and Depression in a Patient with Brugada Syndrome

**DOI:** 10.1155/2014/478397

**Published:** 2014-07-10

**Authors:** Jasper J. Chen, Rajbir S. Sangha

**Affiliations:** ^1^Behavioral Health Services, Cheyenne Regional Medical Center, Cheyenne, WY 82001, USA; ^2^Department of Psychiatry, Geisel School of Medicine at Dartmouth College and Dartmouth-Hitchcock Medical Center, Lebanon, NH 03756, USA; ^3^Department of Cardiology and Electrophysiology, Dartmouth-Hitchcock Medical Center, Lebanon, NH 03756, USA

## Abstract

*Background*. Brugada syndrome is rare and has been a clinically diagnosable entity since 1992. Its clinical manifestations are highly variable, and while some patients remain asymptomatic, others endure sudden cardiac death. Initial presenting symptoms may include palpitations, seizures, syncope, and nocturnal agonal respiration. The diagnosis of Brugada syndrome relies on both clinical findings and characteristic ECG patterns that occur spontaneously or are induced by usage of sodium-channel blocking agents. *Aims of Case Report*. Many psychiatrists may be unaware of the possibility of medical cocontributing etiologies to physical symptoms of anxiety and depression. We present a case of a patient who was treated psychiatrically for anxiety and panic attacks and who was subsequently diagnosed with Brugada syndrome and treated medically with an implantable cardioverter defibrillator (ICD), the only treatment option demonstrated to be effective. Her psychiatric symptoms predated her diagnosis of Brugada syndrome by at least fifteen years. *Conclusion*. The patient's eventual diagnosis of Brugada syndrome altered the course of her psychopharmacologic medication management and illustrates the utility of a psychosomatic approach to psychiatric symptom management.

## 1. Background

Brugada syndrome is a rare autosomal dominant disorder with highly variable clinical manifestations. It results in an increased risk for ventricular arrhythmias; while some patients remain asymptomatic, sudden cardiac death (SCD) can be the initial presentation [1]. Initial presenting symptoms may include palpitations, seizures, syncope, and nocturnal agonal respiration [2].

In approximately 20% of known cases, Brugada syndrome is caused by mutations in the SCN5A gene on chromosome 3p21-23, encoding the alpha-subunit of the cardiac sodium- channel [3]. The point prevalence of the Brugada syndrome electrocardiogram (ECG) pattern is estimated at 1–5 per 10,000 individuals [4]. However, it is difficult to specify the true prevalence of this often underdiagnosed disease because the ECG is often concealed and dynamic [5]. The characteristic ECG pattern shows a QRS morphology similar to right bundle branch block, with ST-segment elevation in leads V1–V3 [5]. Similar to long QT syndrome, some Brugada syndrome patients display a QT prolongation in their ECG, but this is usually limited to the right precordial leads V1–V3 [5].

The prevalence of Brugada syndrome is variable, from 0.4% in the United States to as many as 1% in Asia; this is likely an underestimate because many individuals, in the absence of ECG studies, present with silent forms of the disease [6]. It has been proposed that Brugada syndrome may account for 20% of SCD in the absence of structural heart disease and may be responsible for between 4% and 12% of all patients with SCD [7]. Supraventricular tachycardias (SVTs) have been reported in up to 20% of patients with Brugada syndrome [8]. Arrhythmias and Brugada syndrome symptoms typically appear when there is a preponderance of vagal activity, such as during rest or sleep [9].

The diagnosis of Brugada syndrome relies on both clinical findings and characteristic ECG patterns that occur spontaneously or are induced by usage of sodium-channel blocking agents [10]. The sensitivity of drug challenge can be highly variable. To date, the only effective treatment option is placement of an implantable cardioverter defibrillator (ICD) [11]. However, education and prevention of arrhythmias via lifestyle modification and awareness are also considered [7].

We present the case of a female patient with a significant history of generalized anxiety, panic attacks, and depression which predated her diagnosis of Brugada syndrome by at least fifteen years. We discuss how her eventual medical diagnosis and treatment altered the course of her psychiatric care.

## 2. Case Report

A 46 year-old female patient with a past psychiatric history of anxiety and depression had been taking venlafaxine and trazodone as prescribed by her primary care physician (PCP) for the past ten years. In April of 2013, her neurologist thought that she may have had excess serotonin causing her headaches, so her psychiatric symptoms were then treated with trazodone only. When she stopped her venlafaxine, her headaches stopped and her palpitations stopped as well. Over the last few months prior to psychiatry referral, the patient had been feeling worse and had restarted venlafaxine in July of 2013 to ameliorate her mood, but this again caused her headaches and palpitations to return, and thus the venlafaxine was stopped again.

The patient had recently begun working full time at a daycare which she believed was triggering her mood symptoms. Just two days into her new job, she began feeling very anxious. Her duties required her to move around different rooms each day, which also overwhelmed her. As a result, the patient experienced many episodes of gastrointestinal upset, including diarrhea, bladder and fecal incontinence, and vomiting in addition to episodes of shaking and crying. Triggers for the patient's anxiety attacks included her perception of others' disappointment in her. At times, the patient stuttered and even broke her teeth. The patient stated that “I pee my pants, I get diarrheas [*sic*], and I have to go home because I can't hold it.”

The patient had been psychiatrically hospitalized several times for suicidal ideation (SI), although she denied having any history of suicide attempts. The patient had previously failed multiple medications including fluoxetine (ineffective), paroxetine (caused fatigue), sertraline (caused exacerbation of anxiety and depression), citalopram (caused thoughts of SI and changed mood), or bupropion (caused a seizure). The patient had tried tri-cyclic antidepressants (TCAs) for migraines remotely, but not recently.

Psychiatry was asked to see this patient in the primary care setting as part of an ongoing, developing model of integrated care at Dartmouth-Hitchcock Medical Center. On mental status exam, the patient was in distress, tearful, frequently requiring tissues, and hyperventilating and the patient's speech was appropriate, although talking about work exacerbated her crying. She denied having any suicidal, homicidal, or violent ideation. She was taking escitalopram 10 mg oral daily, alprazolam 0.5 mg oral daily as needed for anxiety, and trazodone 100 mg oral nightly. Family history was notable for a mother with severe depression as well as maternal grandmother with severe depression and alcoholism. The patient lived locally, had raised two sons, and denied using any substances.

We endeavored to assist the patient and her family to improve her functional ability to work. Our review of the patient's prior medication trials prompted the transition from the SSRI back to TCA class, as the patient endorsed previously tolerating a TCA for headache prevention. Given the patient's desire to target improvements in both sleep and gastrointestinal symptoms, she consented to discontinue her escitalopram and trial nortriptyline.

Over the next several months, the patient's symptoms improved. She was able to work and appeared visibly improved on follow-up visits. However, over the next several months she appeared on numerous occasions at her PCP's office and in the emergency department (ED) for what were thought to be syncope episodes. These appeared suddenly without prodrome and would last anywhere from one to ten minutes. She estimated having about a dozen since March 2013 and then started having them twice per week. When she awoke from them, she had dyspnea, palpitations, and grogginess. No clear triggers were identified.

Myriad ED presentations led to “Ziopatch” ambulatory heart rhythm monitoring, which showed sustained paroxysmal ventricular tachycardia (PVT)/ventricular fibrillation (VF) up to 90 seconds associated with her syncope. She was immediately admitted and initiated cardiac workup, including an echocardiogram, left heart catheterization, and cardiac MRI, which were unremarkable. As part of her cardiac electrophysiology evaluation, a drug challenge was performed with 400 mg of oral flecainide. Within an hour of drug challenge, a Type I Brugada ECG response was manifest. The patient also noted symptoms of diaphoresis, palpitations, and nausea similar to her previously unexplained episodes during her drug challenge. These symptoms resolved hours after the flecainide was given and the drug effect had ceased.

Given a Type I Brugada response to flecainide, she was diagnosed with Brugada syndrome. It appeared likely that her nortriptyline had exacerbated this condition. In retrospect, the patient's spouse described episodes consistent with nocturnal agonal respiration, giving added weight to this diagnosis. Given the patient's relatively young age and VT episodes, she underwent subcutaneous ICD placement, which was well-tolerated. Genetic screening was also obtained prior to discharge in light of her two children. She was given specific instructions regarding both her ICD placement regarding care and activity and disease-specific counseling.

## 3. Discussion

We are unaware of any case reports, series, or studies investigating the directionality of mood and anxiety symptoms in Brugada syndrome. The rarity of Brugada syndrome itself makes this type of research inherently difficult. However, it is also unclear to what extent patients presenting with physical symptoms of anxiety—including palpitations, tachycardia, and other distressing symptoms—have a thorough cardiac workup beyond an ECG and/or cardiac enzymes to rule out MI unless syncope or seizures capture the attention of medical teams.

This case illustrates how an underlying medical condition—albeit undiagnosed—can interfere with the use of a medication which would otherwise be an optimal choice for the management of physical symptoms of anxiety and depression. For good reason, current generation psychiatrists are taught during their residencies to be comfortable prescribing TCAs but often as third-line agents following trials of SSRIs, SNRIs, and other medication classes. The basis for the reluctance to use TCAs more often in addressing psychiatric symptoms is well founded in their pharmacologic profile: lethality in overdose and serious side effect profile including arrhythmias, constipation, and dryness.

However, our patient's symptomatology suggested that TCAs were preferred pharmacologic treatments given their anticholinergic effects, which intended to treat diarrhea and fecal incontinence—symptoms preventing her from working. It was also known that the patient had at least tolerated TCAs for some duration in treating her headaches. In hindsight, if the prescribing physician had known that the patient had Brugada syndrome, they would not have entertained the notion of prescribing a TCA. This case report underscores the important teaching that TCAs should be prescribed judiciously and cautiously.

Following the patient's diagnosis, her primary outpatient psychiatrist and cardiologist collaborated to determine optimal psychopharmacologic treatment. We decided to try mirtazapine starting at 7.5 mg PO nightly and titrated to 15 mg PO nightly, which has been well tolerated. The patient also began regular group and individual therapy to learn more coping strategies. Were the patient's mood symptoms to deteriorate, psychiatrists may consider whether or not electroconvulsive therapy (ECT) would be warranted, although there have been case reports describing Brugada syndrome as a potential cardiac risk factor during ECT [12].

Furthermore, all psychiatrists should be aware of sudden cardiac death associated with psychopharmacologic treatment [13]. We have traditionally thought of life-threatening ventricular arrhythmias in the absence of structural heart disease to include Torsades de pointes and/or long QT syndrome. As this case illustrates, we should also consider Brugada syndrome [5]. As ion channelopathies, these syndromes are affected by psychotropic medications.

## 4. Conclusion

Psychiatrists should be aware that psychotropic medications blocking sodium-channels may increase the risk of syncope and sudden cardiac death by triggering Brugada syndrome [14, 15]. These psychopharmacologic agents include carbamazepine/oxcarbazepine, lamotrigine, valproic acid, tricyclic antidepressants, phenothiazines, clozapine, and SSRIs—all of which may interfere with sodium-channel activity.

When individuals treated with psychotropic medications develop concerning cardiac symptoms, Brugada syndrome must be on the differential diagnosis. Therefore, the clinical utility of obtaining ECG in these patients is underscored [5] and involvement of a cardiologist or electrophysiologist may be necessary to confirm the diagnosis. Given the frequency with which patients are treated with mood stabilizers and the fact that Brugada syndrome is infrequently diagnosed, further research on the impact of psychopharmacological medication on Brugada syndrome would improve their safety [5].

The generalizable lesson is to be vigilant in ruling out medical disorders contributing to or exacerbating a patient's psychiatric symptoms. It may be that Brugada syndrome is simply underdiagnosed and relatively unknown. Certainly, most psychiatrists are not aware that Brugada syndrome may be on the differential diagnosis of a patient who presents clinically with physical symptoms of anxiety. It is also inadvisable to prematurely or routinely subject patients to cardiac catheterization and unnecessary procedures, such as ICD implantation, unless clinically warranted. However, as the current research suggests, there is a risk of sudden cardiac death associated with Brugada syndrome, and thus prompt diagnosis and treatment are imperative.
